# The Prognostic Value of the CA19-9/TBIL Ratio in Patients with Biliary Tract Cancers (BTCs): A Retrospective Study

**DOI:** 10.1155/2021/5829893

**Published:** 2021-02-23

**Authors:** Jianchun Xiao, Li Wang, Tao Hong, Binglu Li, Wei Liu, Qiang Qu, Chaoji Zheng, Xiaodong He

**Affiliations:** Department of General Surgery, Peking Union Medical College Hospital, Beijing 100730, China

## Abstract

**Background:**

Incidence of biliary tract cancers (BTCs) in China is high, and surgery is the only curative option. Preoperative CA19-9 has been identified as a predictor for survival in patients with resectable BTCs, but more potential predictors need to be studied. This retrospective study aimed to establish the prognostic significance of CA19-9/TBIL ratio (CTR) in patients with BTCs.

**Methods:**

A retrospective analysis was performed in patients who were diagnosed with BTCs and received surgical resection between 2013 and 2018 at PUMCH. Demographic and clinical parameters were collected. Preoperative CA19-9 and CTR were classified as elevated (>58.6 and >0.83) according to the receiver operating characteristic (ROC) analysis. Demographic and clinical parameters were compared between the groups using Student's *t*-test, chi-square, or Fisher's exact test. Survival analysis was performed by the Kaplan–Meier methods, and the relationship between variables and survival was assessed by the log-rank test. Cox regression analysis was conducted to identify potential risk factors for overall survival.

**Results:**

In total, 109 participants were involved in the final analysis. The overall survival rate was 18.0% at 5 years, with a median survival duration of 1.58 years. The Kaplan–Meier analysis indicated that higher CTR was associated with shorter OS (15 vs. 50, *p* < 0.01). Univariate survival analysis identified TNM staging, CA19-9, and CTR as statistically significant prognostic factors. In a multiple Cox analysis, only CTR was proved as a significantly independent prognostic factor.

**Conclusion:**

CTR acts as an independent prognostic predictor for patients with biliary tract cancer.

## 1. Background

Biliary tract cancers (BTCs) arise from epithelial cells of the biliary duct tree and are the second most common primary hepatobiliary cancer, which can be further subdivided into gallbladder cancer (GBC), perihilar cholangiocarcinoma (PC), distal cholangiocarcinoma (DCC), ampulla of Vater cancer (AVC), and intrahepatic cholangiocarcinoma (IHC) [1, 2]. The incidence of BTCs varies in different regions, and China has a high incidence (>6 cases per 100,000 people) which is increasing over time [3–5]. And surgery remains the only curative option with a low resectability rate [6].

Serum carbohydrate antigen 19-9 (CA19-9) has been widely used as a biomarker in diagnosis, clinical monitoring, and prognosis of BTCs. CA19-9 is a sialylated Lewis blood group antigen, whose levels depend on the Lewis phenotype. Lewis-negative individuals, accounting for 5–10% of the population, have low even absent secretion of CA19-9 even when they have malignant tumors [7, 8]. A CA19-9 level of 5 U/mL has been suggested as the threshold value for Lewis (+) and Lewis (−) population when Lewis genotyping is unavailable [8, 9]. Therefore, patients with a CA19-9 level of <5 U/mL should be excluded.

Moreover, elevated levels of CA19-9 can also be found in patients with benign diseases and affected by jaundice and biliary tract inflammation [10–12]. Adjusted CA19-9 was proposed and applied to improve the power of CA19-9 in the differential diagnosis between malignant and benign jaundice [12, 13], in which CA19-9 was adjusted by dividing it by serum bilirubin level and C-reactive protein (CRP).

Currently, many reports indicate that the preoperative CA19-9 level correlates inversely with survival in patients with resectable BTCs, in which cutoff values of CA19-9 are defined by different levels [14–19]. However, the utility of adjusted CA19-9 is rare in the prognosis of BTCs, and only one study identified elevated preoperative CA19-9/bilirubin ratio as an independent negative prognostic factor after resection of distal cholangiocarcinoma (DCC) based on the German population [20]. This study aims to assess and compare the prognostic value of the preoperative CA19-9 level and CA19-9/TBIL ratio (CTR, bilirubin-adjusted CA19-9) in patients with BTCs based on the Chinese population.

## 2. Methods

### 2.1. Patients

Demographic, clinical, and pathologic tumor characteristics were retrospectively collected from the medical records of 130 patients who were diagnosed with BTCs and received surgical resection from January 2013 to June 2018 at Peking Union Medical College Hospital (PUMCH). The exclusion criteria included the following: (1) patients have no information on preoperative CA19-9 levels and follow-up data; (2) diagnosis of BTCs was not pathologically confirmed; and (3) the value of CA19-9 serum levels < 5 U/mL which was considered as Lewis antigen negative.

The final analysis involved 109 patients who were diagnosed with BTCs, including gallbladder cancer (GBC), ampulla of Vater cancer (AVC), hilar cholangiocarcinoma (HC), distal cholangiocarcinoma (DCC), and intrahepatic cholangiocarcinoma (IC), and treated by surgical resection. The primary endpoint was overall survival (OS), which was defined as the interval between the diagnosis and death or the last follow-up. Complete information about the survival status could be obtained for all patients. Follow-up was performed by conducting telephone interviews or reviewing medical records and ended on August 31, 2019.

### 2.2. Clinical Variables

Clinical variables include demographic data, tumor location, residual tumor stage, degree of pathological classification, TNM staging, jaundice, total bilirubin (TBIL), and CA19-9. Other data were extracted from medical records.

### 2.3. CA19-9 and CA19-9-to-TBIL Ratio (CTR)

CA19-9 and TBIL were assayed within one week before diagnosis. CTR was calculated by dividing CA19-9 by the TBIL: CTR = CA19-9/TBIL, as defined previously [12]. CA19-9 has been widely used in predicting prognosis, but the cutoff values vary among different studies, ranging from 22 to 200 U/mL [14, 16, 18–28]. There is a lack of consensus on the exact cutoff point of CTR. In this study, survival receiver operating characteristic (ROC) analysis was conducted to determine the optimal cutoff values for CA19-9 and CTR (58.6 and 0.83). According to the optimal cutoff values, patients were classified into high-CA19-9 (>58.6) and low-CA19-9 (≤58.6) groups and high-CTR (>0.83) and low-CTR (≤0.83) groups.

### 2.4. Statistical Analysis

Demographic and clinical characteristics were compared between different groups using Student's *t*-test for continuous variables and chi-square or Fisher's exact test for categorical variables. Patients' cumulative survival rates were calculated by the Kaplan–Meier method, and differences between subgroups were assessed by the log-rank test. Cox proportional hazards regression model was performed to identify potential predictors for prognosis, including univariate and multivariate Cox analysis. Variables significant in univariate Cox regression and variables of interests were involved in multiple Cox analysis. All statistical analyses were performed using R version 3.6.3. A *p* value < 0.05 was statistically significant.

## 3. Results

### 3.1. Patients' Characteristics and the Relationship with CTR and CA19-9

A total of 109 patients were involved in the final analysis with 21 patients excluded for not meeting the criteria. The demographic and clinical characteristics of patients are shown in Table 1. The mean age of the involved patients was 62.6 ± 9.7 years, with 57.8% younger than 65 years. The majority of patients included in this study was male (71, 65.1%), without jaundice (71, 65.1%), and with R0 residual tumor stage (63, 57.8%). CA19-9 was elevated (>37 U/mL) in 81.65% of all patients with BTCs, and the overall OS had a median of 19 months.

**Figure 1:**
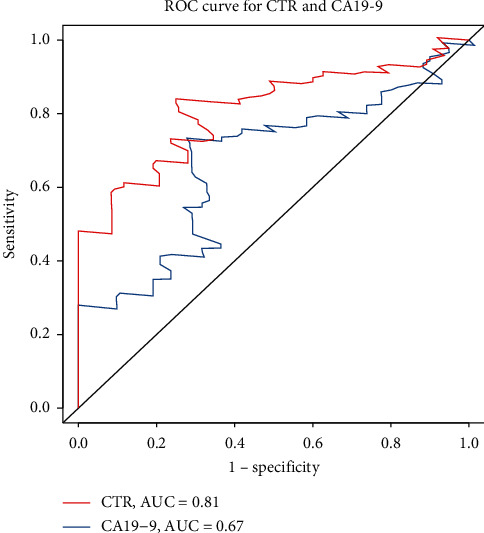
ROC curves and area under the ROC curve for CTR and CA19-9 as a predictor for prognosis of patients with BTCs. CTR: CA19-9/TBIL ratio; BTCs: biliary tract cancers.

Receiver operating characteristic (ROC) curve analysis shows good classification power of CTR in Figure 1 (AUC of CA19-9: 0.67; AUC of CTR: 0.81). According to the ROC, CTR shows good predictive power. ROC curve analysis defines the optimal cutoff value of CTR and CA19-9 as 0.83 and 58.6, which stratifies the whole study population into high-CTR (>0.83) and low-CTR (≤0.83) groups and high-CA19-9 (>58.6) and low-CA19-9 (≤58.6) groups. As shown in Table 1, the demographic and clinical characteristics were similar between the two CTR groups. And the equivalent analysis was also conducted between CA19-9 groups; the result shows no significant differences between the two groups in demographic and clinical characteristics.

### 3.2. Prognostic Significance of CTR and CA19-9

**Figure 2:**
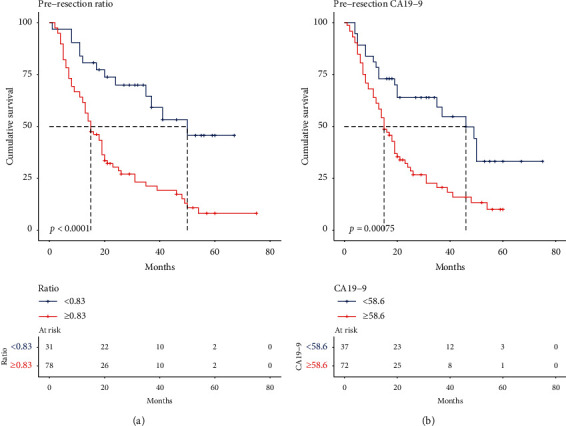
(a) Kaplan–Meier curve comparing overall survival for CTR ≤ 0.83 (blue) and CTR > 0.83 (red), *p* < 0.0001. (b) Kaplan–Meier curve comparing overall survival for CA19-9 ≤ 58.6 (blue) and CA19-9 > 58.6 (red), *p*=0.000075. Vertical ticks represent censored patients, while numbers below the *x*-axis represent the number of patients at risk in each group in 20-month intervals. CTR: CA19-9/TBIL ratio.

The Kaplan–Meier analysis indicated that patients with a CTR >0.83 had worse OS than patients with CTR ≤0.83 (median OS: 50 months vs. 15 months, *p* < 0.0001; Figure 2(a)), and patients with CA19-9 > 58.6 had worse OS than patients with CA19-9 ≤ 58.6 (median OS: 46 months vs. 15 months, *p*=0.00075; Figure 2(b)). 5-year survival rates for patients with a CTR > 0.83 vs. CTR ≤ 0.83 are 8.1% vs. 45.6%, and those for patients with CA19-9 > 58.6 vs. CA19-9 ≤ 58.6 are 10% vs. 33.2%.

### 3.3. Potential Predictive Factors for Prognosis

**Figure 3:**
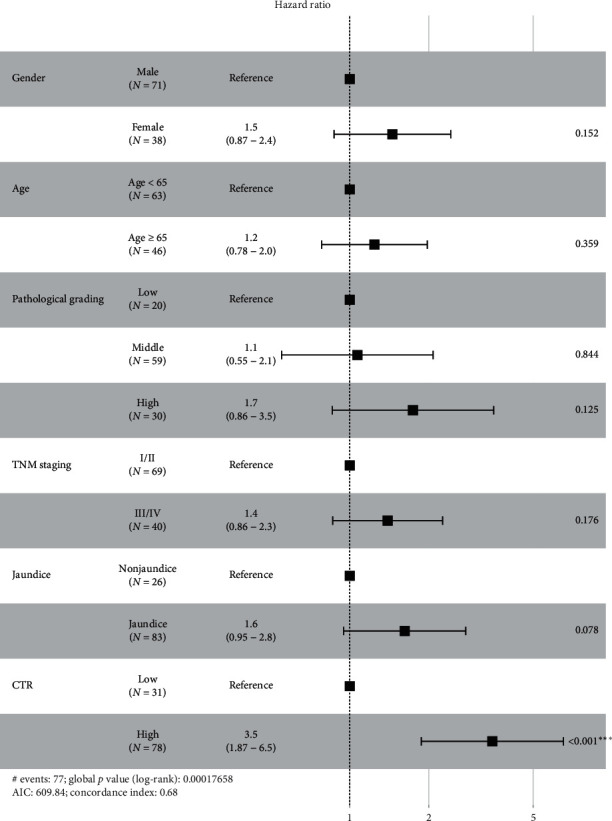
Multivariate Cox regression analysis indicates that CTR is the only significant risk factor independently prognostic for OS. CTR: CA19-9/TBIL ratio.

Cox regression was applied to identify potential significant risk factors for prognosis. In the univariate analysis, age and TBIL were transformed into binary categorical variables with cut points 65 years and 1.5 ULN (33.3 mol/L). TNM staging, CA19-9, and CTR were significant predictors of prognosis as indicated in Table 2. A high CTR was significantly associated with shorter OS (HR = 3.223, 95% CI = 1.766–5.882, *p* < 0.01). These risk factors and other factors of interest were involved in the multiple Cox regression, but CTR >0.83 was the only significant risk factor independently prognostic for OS (HR = 3.5, *p* < 0.01; Figure 3).

## 4. Discussion

Biliary tract cancers (BTCs) are known to have a poor prognosis. Surgical resection is the only potentially curative option for patients with BTCs, but a mere 10–40% of patients present with resectable malignancy [6]. Even for those who received surgical resection, the rates of curative resection vary from 20% to 70% depending on the tumor location, and the recurrence rate is 40–60%, which represents the major reason for low survival rate [14]. It was also reported that advanced BCT patients gained modest survival with only best supportive care (BSC) [29]. Therefore, prognostic tools and preoperative predictive biomarkers are essential to evaluate the survival probability and provide prognostic information for the decision of treatment.

Preoperative CA19-9 showed a negative correlation with survival rate in both BTC patients with no chance for surgery and that after surgery [30], but the reliability of elevated CA19-9 levels is not high enough to reflect the real situation of cholangiocarcinoma for the disturbance of bilirubin, biliary duct inflammation, and other benign diseases [9–11]. A prevalent method to adjust CA19-9 was dividing the preoperative CA19-9 levels by the total bilirubin level (CA19-9/TBIL ratio, CTR), which was adopted by many studies on the prognosis of pancreatic cancer [9, 31–33]. However, CTR was rarely used in prognostic studies of BTCs.

This study constructed a CA19-9/TBIL ratio (CTR) based on preoperative CA19-9 and total bilirubin. Furthermore, we identified CTR as an independent predictor of overall survival time in patients with resectable BTCs. A comparison between preoperative CA19-9 and CTR indicated that CTR served as a better predictor.

CA19-9 has been widely used in predicting prognosis, but the cutoff values vary among different studies, ranging from 22 to 200 U/mL [14, 16, 18, 19, 27]. In this study, cutoff points of CA19-9 and CTR were calculated by survival ROC analysis, and optimal cutoff values for CA19-9 and CTR were 58.6 U/mL and 0.83 (U/mL/*μ*mol/L), which were similar with those of some previous studies [13, 14, 20]. Further large population study is required to determine the uniform standards.

Previous similar studies suggest some common potential significant predictors for the prognosis of BTCs, including preoperative CA19-9, metastasis, pathological grading, residual tumor stage, and jaundice [14, 17–19, 27], but in the present study, univariate analysis indicates that TNM staging (HR = 1.399, *p*=0.04), preoperative CA19-9 (HR = 2.387, *p*=0.001), and CTR (HR = 3.223, *p* < 0.001) serve as significant prognostic factors, ruling out residual tumor stage (*p*=0.6), pathological grading (*p*=0.06), and jaundice (0.8). In another analysis based on 91 patients with BTCs, only elevated preoperative CA19-9 was significantly predictive in univariate and multivariate analyses [16]. The conclusions of different studies are diverse, which may be associated with relatively small sample sizes, different proportions of BTCs, and lack of uniform standards.

This study identified the elevated CTR (>0.83) as the only independent predictor of worse overall survival in patients with BCTs, and CTR is suggested to be applied in future studies on BTC prognosis.

There are several limitations in this study. First, this is a retrospective study, and data were mainly retrieved from electronic medical records and follow-up by telephone. Consequently, missing data and subjective information are inevitable. C-reactive protein (CRP) was also introduced in the method to adjust CA19-9 [12], and metastasis was found significantly prognostic in previous studies [14, 18, 19], but they were not involved in this study. Furthermore, the insufficient sample size and imbalanced subtype proportion of this study limited the prognostic analysis of CTR in each specific subtype of BTCs.

## 5. Conclusion

In summary, this study established the prognostic value of CTR in patients with BTCs based on the Chinese population. The comparison between CTR and preoperative CA19-9 showed that CTR acted better as an independent predictor for overall survival. This study provided an alternative prognostic predictor for BTCs, and further large-population-based study is required to determine uniform standards.

## Figures and Tables

**Table 1:** Patients' demographic and preoperative clinical characteristics.

Variables	CTR ≤ 0.83	CTR > 0.83	Total	*p* value
*Age (mean, SD, n* = *109)*	59.8 ± 11.7	63.8 ± 8.6	62.6 ± 9.7	0.052

*Gender (n, %)*				
Male	21 (65.6%)	50 (64.9%)	71 (65.1%)	0.945
Female	11 (34.4%)	27 (35.1%)	38 (34.9%)	

*Tumor location (n, %)*				0.517
Hilus	9 (29%)	23 (29.5%)	32 (29.4%)	
Gallbladder	0 (0%)	1 (1.3%)	1 (0.9%)	
Common bile duct	17 (54.8%)	39 (50.0%)	56 (51.4%)	
Ampulla	5 (16.1%)	9 (11.5%)	14 (12.8%)	
Intrahepatic	0 (3.1%)	6 (7.7%)	6 (5.5%)	

*TNM staging (n, %)*				0.073
0	2 (6.5%)	0 (0%)	2 (1.8%)	
I	11 (35.5%)	18 (23.1%)	29 (26.6%)	
II	8 (25.8%)	30 (38.5%)	38 (34.9%)	
III	10 (32.3%)	27 (34.6%)	37 (33.9%)	
IV	0 (0%)	3 (3.8%)	3 (2.8%)	

*Residual tumor stage (n, %)*				0.438
R0	24 (77.4%)	57 (73.1%)	81 (74.3%)	
R1	7 (22.6%)	17 (21.8%)	24 (22%)	
R2	0 (0%)	4 (5.1%)	4 (3.7%)	

*Pathological grading (n, %)*				0.184
Low grade	9 (29.0%)	11 (14.1%)	20 (18.3%)	
Middle grade	14 (45.2%)	45 (57.7%)	59 (54.1%)	
High grade	8 (25.8%)	22 (28.2%)	30 (27.5%)	

*Jaundice (n, %)*				0.149
Yes	27 (87.1%)	56 (71.8%)	83 (76.1%)	
No	4 (12.9%)	22 (28.2%)	26 (23.9%)	

CTR: CA19-9/TBIL ratio.

**Table 2:** Univariate Cox regression analysis (*n* = 109).

Variables	HR (95% CI)	*p* value
Gender (female)	1.251 (0.770–2.033)	0.365
Age (>65)	1.330 (0.847–2.088)	0.215
Residual tumor stage (R1/R2)	1.118 (0.664–1.882)	0.674
Pathological grading (middle)	1.420 (0.746–2.701)	0.286
Pathological grading (high)	1.942 (0.964–3.911)	0.063
TNM staging (III/IV)	1.399 (1.005–1.946)	0.040^*∗*^
Jaundice	1.063 (0.609–1.855)	0.800
CA19-9	2.387 (1.413–4.033)	0.001^*∗*^
CTR	3.223 (1.766–5.882)	<0.001

CTR: CA19-9/TBIL ratio.

## Data Availability

No data were used to support this study.
